# Reliability of neural food cue-reactivity in participants with obesity undergoing bariatric surgery: a 26-week longitudinal fMRI study

**DOI:** 10.1007/s00406-020-01218-8

**Published:** 2020-12-17

**Authors:** Patrick Bach, Martin Grosshans, Anne Koopmann, Peter Kienle, Georgi Vassilev, Mirko Otto, J. Malte Bumb, Falk Kiefer

**Affiliations:** 1 Department of Addictive Behavior and Addiction Medicine, Medical Faculty Mannheim, Central Institute of Mental Health, Heidelberg University, J5/68159 Mannheim, Germany; 2 Feuerlein Center on Translational Addiction Medicine (FCTS), University of Heidelberg, Heidelberg, Germany; 3 Department of Surgery, Theresienkrankenhaus, Mannheim, Germany; 4 Department of Surgery, University Medical Center Mannheim, Heidelberg University, Mannheim, Germany

**Keywords:** Food cue-reactivity, Reliability, Intraclass correlation, fMRI, Dice, Jaccard

## Abstract

Obesity is highly prevalent worldwide and results in a high disease burden. The efforts to monitor and predict treatment outcome in participants with obesity using functional magnetic resonance imaging (fMRI) depends on the reliability of the investigated task-fMRI brain activation. To date, no study has investigated whole-brain reliability of neural food cue-reactivity. To close this gap, we analyzed the longitudinal reliability of an established food cue-reactivity task. Longitudinal reliability of neural food-cue-induced brain activation and subjective food craving ratings over three fMRI sessions (T0: 2 weeks before surgery, T1: 8 weeks and T2: 24 weeks after surgery) were investigated in *N* = 11 participants with obesity. We computed an array of established reliability estimates, including the intraclass correlation (ICC), the Dice and Jaccard coefficients and similarity of brain activation maps. The data indicated good reliability (ICC > 0.6) of subjective food craving ratings over 26 weeks and excellent reliability (ICC > 0.75) of brain activation signals for the contrast of interest (food > neutral) in the caudate, putamen, thalamus, middle cingulum, inferior, middle and superior occipital gyri, and middle and superior temporal gyri and cunei. Using similarity estimates, it was possible to re-identify individuals based on their neural activation maps (73%) with a fading degree of accuracy, when comparing fMRI sessions further apart. The results show excellent reliability of task-fMRI neural brain activation in several brain regions. Current data suggest that fMRI-based measures might indeed be suitable to monitor and predict treatment outcome in participants with obesity undergoing bariatric surgery.

**Supplementary Information:**

The online version contains supplementary material available at 10.1007/s00406-020-01218-8.

## Introduction

Obesity affects more than 650 million people worldwide [43]. Overweight has been identified as a major cause of cardiovascular diseases, diabetes, musculoskeletal disorders as well as several types of cancer [5]. The assessment of behavioral and neural responses towards food cues has received some interest in the last decade as a tool to investigate the neurobiological basis of obesity [18, 26]. A recent meta-analysis on food cue-reactivity concluded that across 45 published reports the overall effect of food cue-reactivity and craving on outcomes in patients was of medium size (*r* = 0.3) with a large variability across studies. Authors concluded that food cue exposure and the experience of craving have a significant influence on and contribute to eating behavior and weight gain [6]. Functional magnetic resonance imaging (fMRI) was used to identify the neural correlates of food craving, food perception, and food intake. Structures implicated in food‐intake regulation include the anterior insula, inferior frontal and orbitofrontal cortices, the medial temporal cortex with the amygdala and parahippocampus, as well as the nucleus accumbens, and visual cortices [2]. In the last years, efforts were undertaken to establish neural predictors for eating behavior and treatment response. Some studies reported significant associations between neural responses to food cues and weight loss during treatment, but overall, the studies report heterogeneous findings [22]. The inconsistencies in study results demand for an investigation of the reliability and robustness of the applied food cue-reactivity task, because the possibility to establishing meaningful and robust associations between neural brain responses during food cue presentation and any behavioral or clinical variable critically depends on the reliability of the investigated task-fMRI brain activation. Previous studies have demonstrated substantial variability in findings of food cue-reactivity studies. Although brain responses to visual food cues in participants with obesity have been found to have relatively good mean-level reproducibility, they had poor within-subject test–retest reliability. Several factors were associated with the heterogeneity in findings, including different expression of the fat mass and obesity-associated genes (e.g. FTO) [25, 36, 44], fasted state vs. glucose ingestion prior to fMRI [20] and divergent characteristics of the individual study designs, including the structure, timing and stimuli used during the food cue-reactivity fMRI task. Furthermore, there are clear individual differences in food preferences that were associated with additional variance across studies [42]. Additionally, small sample sizes and a lack of power were related to inconsistencies between studies [8]. Moreover, a study comparing the results of 70 different teams analyzing the same dataset, revealed significant variability in the analysis of the same fMRI food cue-reactivity dataset depending on the researchers decision to use a certain the statistical software (e.g. SPM vs. FSL vs. AFNI) or statistical method (parametric vs. non-parametric) as well as the applied smoothing kernel [7]. The results highlight the need for better standardization of the food stimuli and fMRI task designs and the additional data that are collected on participant’s state (hunger, mood, hormones etc.) and personal characteristics that may be used to control for confounding effects in the analyses. The aforementioned findings emphasize the importance of establishing standardized food cue-reactivity paradigms, study protocols and analysis workflows. To this end, guidelines for good practice in food cue-reactivity neuroimaging studies were proposed. According to these guidelines, researchers planning fMRI studies should take special care to: power calculation, hunger state and related factors, personal characteristics, the selection of food-related stimuli, setting well-considered statistical thresholds for whole-brain analyses, minimizing the risk of movement artifacts, analysis of prospective designs as well as predictive modelling. Moreover, the authors suggest to pre-register planned studies and to share the data obtained [40]. In doing so, it would be possible to ensure reproducibility of results across cue-reactivity studies [40].

To date, there is no study that investigated whole-brain reliability of food cue-induced brain activation. To our knowledge, only a single fMRI study investigated the longitudinal reliability of extracted mean brain activation during food cue processing over a mean period of 18 days (3–35 days), which is short considering follow-up periods of clinical studies that run over months. Additionally, reliability was only assessed in a selected range of a priori defined regions of interest (bilateral insula, amygdala, orbitofrontal cortex, caudate and putamen) [9]. The authors reported that in their dataset, only the left orbitofrontal cortex response showed fair reliability, while all other regions of interest showed poor reliability. The authors also stated that the large inter-individual range of days between the two assessment sessions might have limited reliability in their study. Additionally, previous research highlighted that low reliability in fMRI studies might also be associated to the computation of difference scores or difference contrasts, where one condition is subtracted from the other. For example, regarding the food cue-reactivity tasks, it is common to subtract the brain activation during food picture blocks from activation during neutral picture blocks. However, in the case of a high correlation between the constituting conditions of a difference score, the resulting reliability of that score is limited, because much of the shared “true” variance is removed, while the measurement errors are added [23, 35]. To date, however, no study investigated whole-brain reliability of food-cue-induced brain responses over a longer period of time and, importantly, no study to date investigated reliability in samples of patients undergoing surgery. This, however, seems relevant to the ongoing efforts to establish predictors and biomarkers for treatment efficacy in obesity. In this context, it is necessary to determine the reliability of food cue-reactivity in clinical populations undergoing treatment, because only this way the robustness and suitability of cue-reactivity as a biomarker in obesity can be assessed. Hence, we conducted our analyses in a clinical population undergoing surgery, as this sample reflects a sample for whom biomarkers should be established to predict and monitor treatment outcomes using fMRI biomarkers. Hence, we set out to assess the reliability of neural food cue-reactivity in a longitudinal dataset of individuals with obesity over three neuroimaging assessments that were scheduled 2 weeks before bariatric surgery, and 8 and 24 weeks after surgical intervention. We used an unrestricted whole-brain approach and a set of complementary measures for fMRI reliability, aimed at determining the global and local reliability of the difference contrast (food-neutral) and of the constituting food and neutral picture conditions. Additionally, we compared the reliability of food cue-reactivity to the reliability of commonly applied subjective craving measures that were measured during the fMRI session.

## Subjects and methods

### Participants

Current analyses were conducted on a dataset of *N* = 11 individuals with obesity of whom fMRI task data was available for three time points and that were part of a larger longitudinal clinical study, including a total of *N* = 26 participants with obesity, of whom, however, only the *N* = 11 participants met the inclusions criteria for undergoing fMRI scanning (e.g. absence of metal implants, claustrophobia and waist circumference < 160 cm (due to the scanner diameter). The clinical data of the of the whole study group are reported elsewhere. In short, patients showed a percent total weight loss after surgery (%TWL) from T0 to T2 of 23.8%TWL after Roux-en-Y gastric bypass (*n* = 21) and 12.7%TWL after sleeve gastrectomy (*n* = 5) with no significant difference between both procedures (*p* = 0.126). There were also significant reductions of resting heart rate, fasting plasma glucose levels and depressive symptoms (all *p* < 0.001). Only individuals with obesity that already decided to receive bariatric surgery were recruited for this study. The study procedure was approved by the local ethics committee and all participants provided written informed consent.

Individuals with obesity undergoing fMRI had to meet the following inclusion criteria: (i) age between 18 and 65 years, (ii) BMI (kg/m^2^) > 35 (i.e. ≥ grade 2 obesity), (iii) a waist circumference < 160 cm (limited by scanner diameter), (iv) the capacity to give informed consent, (v) no history or current diagnosis of any psychiatric, neurological, neoplastic or untreated endocrine illnesses (with the exception of nicotine addiction), and no current intake of any centrally acting psychoactive or anti-obesity medications (i.e. sedatives, antipsychotics, including long-acting injectable antipsychotics, antidepressants, opioid analgesics as well as DPP (dipeptidyl peptidose IV) inhibitors and GLP (Glucagon-like peptide)-1 antagonists, (vi) all participants with a history of surgical interventions in the gastrointestinal system or contraindications to fMRI scanning (e.g. metal implants), and pregnant or breast-feeding females were excluded.

Twenty-six individuals (17 females and 9 males, mean age 41 ± 12 years, mean BMI 46 ± 6 kg/m^2^) were eligible for analyses (demographics, bariatric surgery, blood analyses as well as behavioral data) and included in the study. Of these 26 individuals, 21 received Roux-en-Y gastric bypass and 5 sleeve gastrectomy. Imaging data could be obtained for 11 obese individuals (10 individuals received Roux-en-Y gastric bypass and 1 sleeve gastrectomy; 15 individuals had to be excluded due to the fact that they did not fit the scanner.

### Procedures

#### T0 (Two weeks before bariatric surgery)

During the first assessment session, sociodemographic data, information on internal and neurological disorders, as well as information on eating habits was collected. In addition, participants were screened for any psychiatric comorbidities using the Structured Clinical Interviews for DSM-IV, SKID-I, [45]. Additionally, a urine drug screening, and in females a pregnancy test was conducted.

fMRI scanning was performed between noon and 3 PM. All participants received a standardized breakfast of 500 kcal (2093 kJ) 6 h before fMRI scanning and did not eat until the scanning. Subsequently, participants completed a series of questionnaires including the Beck Depression Inventory (BDI, [1], the Fagerstrom Test for Cigarette Dependence (FTCD [12] as well as the Yale Food Addiction Scale (YFAS) [16].

#### T1 and T2 (Eight and 24 weeks after bariatric surgery)

At both time points, participants were examined medically, urine drug screenings, and in females a pregnancy test were performed. Moreover, possible changes in medication were documented. MRI measurements were performed at both time points using the same procedures and tasks as during the first scanning session.

### Imaging procedure

#### fMRI food cue-reactivity task

All patients included in the current analyses underwent three different imaging sessions. During these sessions, patients laid in the scanner wearing MRI-compatible goggles, on which sets of visual food and neutral stimuli were presented using a block design. The task consisted of a total of 18 blocks of food stimuli and 12 blocks of neutral stimuli. Each block comprised of a series of five food or neutral pictures. Food stimuli were further divided in three categories: salty high-calorie, sweet high-calorie, low-calorie, yielding six blocks for each category. All stimuli were shown for 4 s (i.e. 20 s per block) in a pseudo-randomized order. Participants were instructed to closely watch each picture and were informed that they will be asked to rate their subjective craving. In-between each picture block, patients were asked to rate their current craving for food on a visual analogue scale (VAS) that ranged from 0—“very weak” to 100—“very strong”. The fMRI took 18 min. Food stimuli chosen were rated according to their ability to induce food craving by 44 voluntary participants at our institution [17] and neutral cues were taken from the International Affective Picture Series [28].

#### fMRI acquisition and pre-processing

A total of 453 images T2*-weighted, echo planar images covering the entire brain were acquired during the food cue task using a 3-T whole-body tomography scanner (MAGNETOM Trio with TIM technology; Siemens). Imaging parameters were: repetition time = 2.41 s, echo time = 25 ms, flip angle = 80°, number of slices = 42, slice thickness = 2 mm, voxel-gap = 1 mm, voxel dimensions = 3 × 3 × 3 mm^3^, field of view = 192 × 192 mm^2^, in-plane resolution = 64 × 64. The short echo time and the 30° flip angle to anterior commissure–posterior commissure orientation was chosen to minimize susceptibility artefacts. Stimuli were presented using Presentation software (version 9.9, Neurobehavioral Systems Inc.) and MRI-compatible goggles (MRI Audio/Video Systems; Resonance Technology Inc., CA).

Functional-imaging data were processed and analyzed using SPM8 and SPM12. The first five scans were excluded from imaging analyses to avoid any artefacts caused by the effects of magnetic saturation. All images were realigned spatially (movement was considered excessive with > 2 mm translation or > 2° rotation), normalized to a standardized EPI template from MNI (Montreal Neurological Institute, Quebec, Canada), and smoothed using an isotropic Gaussian kernel for group analyses (full width at half maximum: 8 mm).

Food cue-reactivity imaging data were analyzed by modelling the different task conditions (food with the subcategories salty high-calorie, sweet high-calorie, low-calorie and neutral) as explanatory variables within a general linear model in SPM implementing the movement parameters as nuisance variables. Individual contrast images (food cues > neutral cues) were computed for each individual and then included into following second-level analyses in SPM. Nicotine consumption (categorical) was considered as covariate, because previous work indicated that nicotine modulates food cue-reactivity [27]. To satisfy a family-wise error rate correction of pFWE < 0.05, we determined a combined height (*p* < 0.001) and extent (*k* ≥ 103) threshold by running 10.000 Monte Carlo simulations using AlphaSim as implemented in the Neuroelf analysis package (www.neuroelf.net) [4], (estimated smoothness was *x*/*y*/*z* = 10.13/9.86/10.33 mm) [11].

### Reliability analyses

We investigated the reliability of subjective food craving ratings (i.e. mean craving for food–mean craving for neutral stimuli during the 1st, 2nd and 3rd assessment session to correspond to the fMRI task contrast “food–neutral”) over the three fMRI sessions by computing the intraclass correlation coefficients using a two-way, mixed effects model in IBM SPSS (version 25.0). Additionally, whole-brain longitudinal reliability of individual brain responses to food stimuli over the three imaging sessions by computing measures of local and global reliability using the fmreli toolbox for SPM12 by Kroemer, Frohner and colleagues [15] (https://github.com/nkroemer/reliability). Analyses were conducted on the whole brain without a-priori restrictions to specific regions of interest.

#### Jaccard and Dice coefficients

We computed the modified Jaccard coefficient, a common measure in fMRI reliability studies between the three different time points for the difference contrast food > neutral and the constituting contrasts (i.e. food and neutral separately). It is defined as the size of the intersection divided by the size of the union of the voxel sets and computed as follows:

$${\text{Jaccard}}\left( {A, B} \right) = \frac{{\left| {A \cap B} \right|}}{{\left| {A \cup B} \right|}} = \frac{{\left| {A \cap B} \right|}}{{\left| A \right| + \left| B \right| - \left| {A \cap B} \right|}}$$

The Jaccard coefficient can be interpreted as the percentage of overlapping significant voxels above a predefined statistical threshold (e.g. *p* < 0.001) within all significant voxels [24, 31].

Additionally, we computed the Dice coefficient for the three different contrasts and scanning time points. It is calculated as the number of super-threshold voxels that overlap between sessions divided by the average number of significant voxels across sessions:

$${\text{Dice}}\left( {A, B} \right) = \frac{{2\left| {A \cap B} \right|}}{\left| A \right| + \left| B \right|}$$

The Dice coefficient was introduced to assess the overlap of significant fMRI clusters between scans. It has become an established measure of fMRI data reliability [37]. Both coefficients have values from 0 (“no overlap”) to 1 (“perfect overlap”) between significant super-threshold voxels. Both measures are, however, limited by the missing consensus on specific values or cut-offs that would differentiate between “poor” and “good” values [3]. Additionally, the magnitude of both coefficients depends on the statistical threshold used to define what is “active”. Studies showed that the reliability of the cluster overlap method decreases, when the significance threshold is increased [10, 38]. In the current analyses, we, therefore, applied a commonly used threshold of *p* < 0.001. Resulting values were imported into the IBM SPSS Statistics software (version 25.0) for further analyses using a repeated measure analysis of variance (ANOVA) model with the factors time (1st, 2nd, 3rd) assessment and task contrast (food, neutral, food > neutral).

#### Similarity

Second, we calculated the within- and between-subject similarity of the fMRI activation maps using the fmreli toolbox [15]. Similarity in this context is defined as the resemblance of two activation patterns based on the alignment of high versus low brain activation values across the brain between- and within-subjects (for details see Frohner et al. 2019). The resulting coefficients are correlation coefficients that range from ‘perfect’ inverse relationship (− 1.00) to a ‘perfect’ direct relationship (1.00). It was suggested that individuals can be successfully identified by their neural activation patterns, if the within-subject similarity exceeds all between-subject association coefficients of the same participant [13, 15]. An advantage of this procedure is that it does not require an a-priori (and potentially arbitrary) statistical threshold.

#### Intraclass correlation (ICC)

Third, we estimated voxel-wise reliability of brain activation patterns by computing the intraclass correlation (ICC) coefficients between all three fMRI sessions. The ICC is used to assess whether the magnitude of activation in each voxel of the brain is stable from test scan to retest scan. Previous work suggested that this measure might be more stringent than other fMRI reliability measures, as it also requires near zero values to be stable over time [3]. It was suggested that the ICC(3,1) variant is most appropriate for assessing longitudinal fMRI datasets [34]. Mathematically, this coefficient sets within-subject variance (σ^2^_within_) in relation to between-subject variance (σ^2^_between_). We used the ICC(3,1)-type to assess voxel-wise reliability [39], defined as:

$${\text{ICC}} = \frac{{\left( {{\upsigma }^{2}_{{{\text{between}}}} - {\upsigma }^{2}_{{{\text{within}}}} } \right)}}{{\left( {{\upsigma }^{2}_{{{\text{between}}}} + {\upsigma }^{2}_{{{\text{within}}}} } \right)}}.$$

According to Fleiss (1986), ICC coefficients lower than 0.4 represent poor reliability, ICCs between 0.4 and 0.75 represent fair (< 0.6)-to-good (> 0.6) reliability, and ICCs higher than 0.75 represent good-to-excellent reliability [14]. We calculated ICC coefficients for every brain voxel to allow identification of brain regions that show high reliability without restriction to predefined regions of interest. However, we were aware that much of the (un-thresholded) brain activation might be unrelated to food cue task and hence would not replicate in its magnitude, resulting in a low overall ICC value. Therefore, we generated thresholded ICC brain maps, to identify brain areas that show good-to-excellent (ICC > 0.75) reliability and we computed additional atlas-based mean ICC values for a standard set of anatomical brain regions (see below).

#### Spearman’s correlation

To assess whether reliability of the common difference contrast food > neutral might be limited by a high correlation between the constituting conditions, we computed the voxel-wise Spearman’s correlation coefficients between the three food image categories (i.e. sweet, high caloric, low caloric) and the neutral condition using the fmreli toolbox.

### Computation of atlas-based summary measures

In accordance to previous work [15], we computed the mean ICC for *N* = 120 anatomical regions specified in the Automatic Anatomic Labeling (AAL) atlas [41]. The additional atlas-based summary intended to facilitate the assessment of local differences in reliability and identify reliable anatomical ROIs for future analyses. ICC values were extracted using the ROI data extraction routine of the MarsBar software package (http://marsbar.sourceforge.net/) and was imported into SPSS (IBM SPSS Statistics version 25.0) for further analyses.

### Group-level fRMI task activation

On a group level, imaging data for every single time point (e.g. 1st, 2nd, 3rd assessment) were analyzed using a one-sample *t* test to assess the robustness of task main effects (i.e. between condition effects) on group-level brain activation and to determine brain areas that show higher brain activation in response to food cues, compared to neutral cues (contrast: food–neutral). Additionally, we performed analyses of changes in food cue-induced brain responses over time, by setting up a flexible factorial model with the within subject factor time (i.e. 1st, 2nd, 3rd assessment) and the covariates BMI at baseline, surgery type and smoking status. In order to satisfy a family-wise error rate correction of *p*FWE < 0.05, we determined a combined voxel-wise- [*p* < 0.001] and cluster-extent-threshold [*k* ≥ 103] by running 10.000 permutations by Monte Carlo simulations (the estimated smoothness was *x*/*y*/*z* = 10.13/9.86/10.33 mm) using the Neuroelf analysis package (www.neuroelf.net) (Bennett et al. 2009) [4].

## Results

### Sample characteristics

Demographical, clinical and psychometric data are depicted in Table 1.

**Table 1:** Demographic and clinical characteristics of obese study participants that underwent three imaging assessments at T0 = 2 weeks prior to surgery, T1 = 8 weeks after surgery and T2 = 24 weeks after surgery (*N* = 11)

*N* = 11 participants with obesity	Absolute numbers	Relative proportions (%)
Sex (male/female)	3/8	27.3/72.7
Smoking status (non-smoking/ < 10 cig. per day/ > = 10 cig. per day)	7/2/2	63.6/18.2/18.2

	Mean	SD
Age (years)	41.18	10.1
Height (m)	1.68	0.1
Weight (kg)	128.78	17.1
BMI	45.40	4.7
BDI (total score)	9.45	4.6

### Group-level food cue-induced brain activation

Group-level analyses of brain activation demonstrated significant food cue-induced brain activation (contrast: food > neutral) in parts of the frontal and orbitofrontal cortex, the occipital and parietal gyri, the cuneus, calcarine, the lingual gyrus, as well as the caudate, putamen, thalamus and insula (see Table 2). On the other hand, no significant brain activation was detected during presentation of neutral pictures compared to food pictures (contrast: neutral > food). Whole analyses of longitudinal changes in brain responses towards food cues over assessment sessions before and after surgery showed no main effect of time on brain response towards food cues and post-hoc comparisons between separate assessment time points did not surpass the predefined statistical threshold.

**Table 2:** Brain depicting higher brain response to visual food cues compared to neutral cues (contrast: food > neutral, combined voxel-wise- [*p* < .001] and cluster-extent-threshold [*k* > 103 voxel], corresponding to pFWE < .05)

Side	Lobe	Brain areas	Cluster size (voxel)	MNI coordinates (*x*, *y*, *z*)			*t*_max_
R and L	Occipital	Superior, Middle and Inferior Occipital Gyrus, Calcarine, Cuneus, Fusiform Gyrus, Lingual Gyrus	7081	32	− 76	− 14	21.9
R	Parietal	Inferior Parietal Gyrus, Angular Gyrus	133	32	− 68	54	9.6
L	Occipital, Parietal	Superior and Middle Parietal and Occipital Gyrus	275	− 24	− 60	44	8.9
L		Putamen, Insula	129	− 40	− 6	10	8.7
L	Parietal	Inferior Parietal Gyrus, Postcentral Gyrus, Supramarginal Gyrus	142	− 48	− 24	40	8.6
R and L		Anterior and Middle Cingulate Gyrus	176	− 8	24	24	7.4
L	Frontal	Middle and Inferior Frontal Gyrus, Orbitofrontal Cortex	130	− 44	36	14	7.2
R		Caudate, Thalamus	104	14	− 4	12	6.9

### Reliability analyses

#### Food craving ratings

Analyses indicated good reliability of the mean subjective food craving ratings during fMRI across the different assessment sessions (ICC [1, 3] = 0.611, *p* = 0.002). Food cues induced higher craving values compared to neutral cues throughout all three assessment sessions. There was a significant reduction in the magnitude of reported food craving over the trial period from baseline (*M* = 45.195, SD = 23.443) to T1 (*M* = 18.550, SD = 39.917) that remained stable until T2 (*M* = 32.450, SD = 25.972, *F*_(2,18)_ = 4.301, *p* = 0.032).

#### Jaccard coefficient

Mean Jaccard coefficients for the comparisons of the different time points are displayed in Table 3. Repeated measures ANOVA showed a significant main effect of contrast image category (neutral, food and food > neutral) (*F*_(2,20)_ = 83.806 *p* < 0.001) on the magnitude of the Jaccard indices. Post hoc analyses demonstrated lower Jaccard coefficients for the difference contrast condition (food > neutral) compared to both constituting conditions (food and neutral, *p* < 0.001). There was no main effect of time on the magnitude of the Jaccard coefficients (i.e. whether we compared to 1st to 2nd or 3rd scanning session, *F*_(2,20)_ = 0.152 *p* = 0.860).

**Table 3:** (A) Dice and (B) Jaccard coefficients for the three task contrasts (food > neutral, food and neutral), illustrating the proportion of overlapping significant voxels between the different fMRI sessions at T0 = two weeks prior to surgery, T1 = eight weeks after surgery and T2 = twenty-four weeks after surgery (whole-brain threshold of *p* < 0.001 for defining super-threshold activation)

Comparison of sessions	Session 1 and 2			Session 1 and 3			Session 2 and 3		
(a) Dice coefficients									
Contrast	Food > Neutral	Food	Neutral	Food > Neutral	Food	Neutral	Food > Neutral	Food	Neutral
Mean	0.2743***	0.6763	0.7103	0.2049***	0.7181	0.7260	0.2218***	0.6921	0.6790
SD	0.2036	0.2160	0.2067	0.1599	0.0763	0.0762	0.1918	0.2187	0.2106
(b) Jaccard coefficients									
Contrast	Food > Neutral	Food	Neutral	Food > Neutral	Food	Neutral	Food > Neutral	Food	Neutral
Mean	0.1744***	0.5400	0.5772	0.1222***	0.5651	0.5750	0.1375***	0.5596	0.5406
SD	0.1443	0.1970	0.1830	0.0996	0.0911	0.0935	0.1316	0.2024	0.1853

*SD* standard deviation

***Significant difference at *p* < 0.001 between the contrast condition food > neutral and each of the other two conditions (food and neutral)

#### Dice coefficient

The mean Dice coefficients for the comparisons of the different fMRI sessions are depicted in Table 3. Analyses demonstrated a significant main effect of contrast image category (neutral, food and food > neutral) (*F*_(2,20)_ = 77.102 *p* < 0.001) on the magnitude of the Jaccard indices. Post hoc analyses demonstrated lower Jaccard coefficients for the difference contrast condition (food > neutral) compared to both constituting conditions (food and neutral, *p* < 0.001). There was no main effect of time on the magnitude of the Jaccard coefficients (i.e. whether we compared to 1st to 2nd or 3rd scanning session, *F*_(2,20)_ = 0.208 *p* = 0.814).

#### ICC

**Fig. 1:**
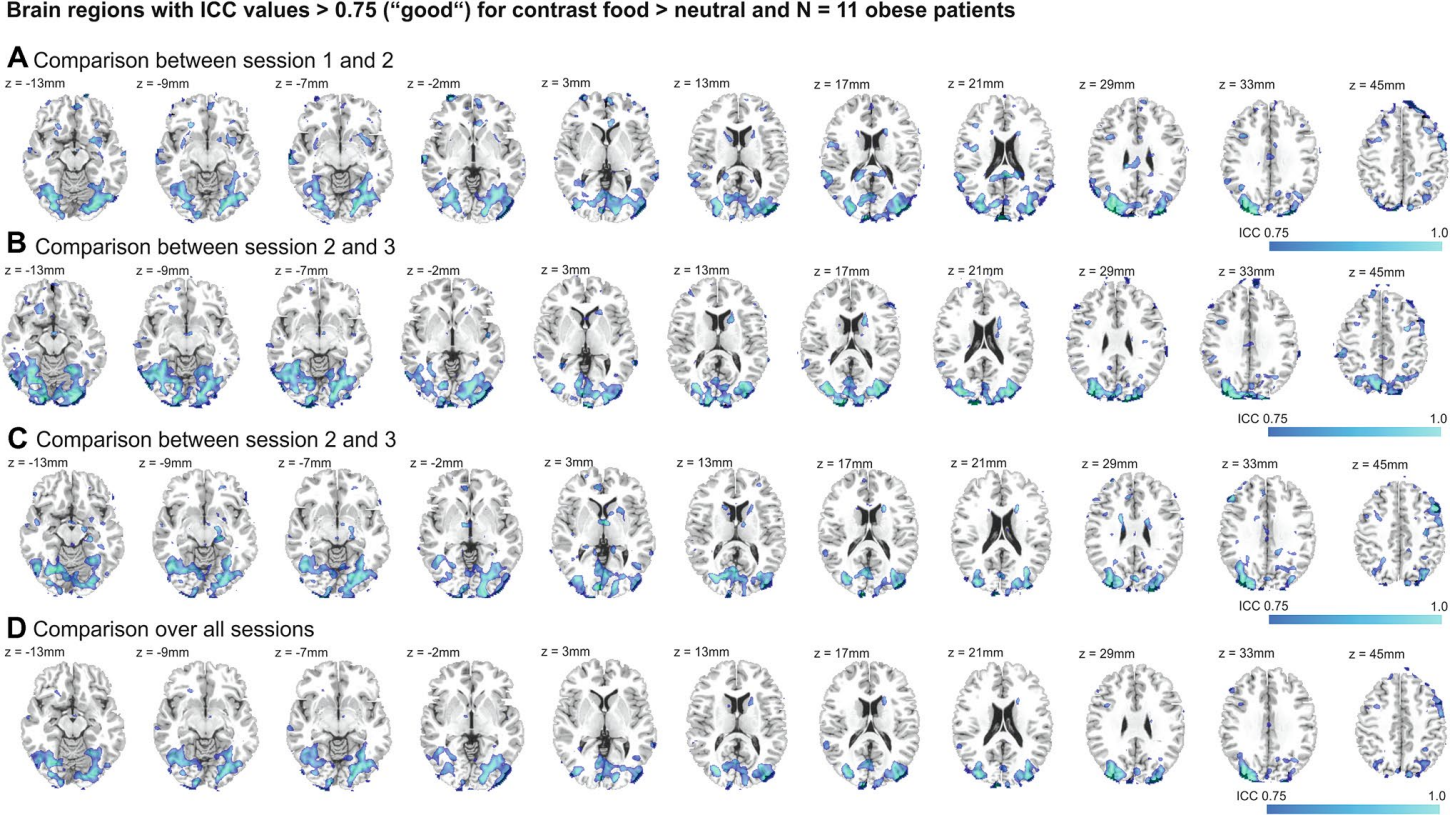
Depiction of brain areas that show good to excellent reliability for the difference contrast food-neutral (Intraclass correlation [ICC] > 0.75) for the comparisons between: **a** session 1 and 2 (i.e. 2 weeks prior to surgery and 8 weeks after surgery), **b** session two and three (i.e. 2 weeks prior to surgery and 24 weeks after surgery), **c** session 1 and three and **d** over all sessions

Comparisons of ICC coefficients between the different fMRI sessions (1st, 2nd, 3rd) indicated that several regions showed good to excellent reliability (i.e. ICC > 0.75) across all sessions (see Fig. 1). These regions included the bilateral caudate and left putamen, parts of the right thalamus and middle cingulum, as well as parts of the bilateral inferior, middle and superior occipital gyri (brodmann areas BA 7/17/18/19/39) and parts of the bilateral middle and superior temporal gyri (BA 20/21/22/37) and in addition parts of the bilateral cunei, lingual gyri and calcarine (see Fig. 1). These patterns appeared to be relatively stable across all session time points, supporting the stability of the observed findings.

In a second step, we determined the mean ICC for a standard set of *n* = 120 anatomical regions of interest defined in the aal atlas. As expected based on the patterns of voxel-wise ICC values (i.e. good to excellent reliability only in parts of the anatomical region), the mean overall ICC for the separate regions did not exceed the voxel-wise values. However, several anatomical regions of interest masks showed good or fair reliability (see supplementary Table S1), specifically the bilateral inferior, middle and superior occipital gyri ROIs showed good overall reliability (> 0.6) and the several other regions showed fair reliability (> 0.4) Left putamen, bilateral caudate, left amygdala, bilateral lingual gyri, right fusiform gyrus, bilateral calcarine, bilateral cunei, posterior cingulate, right middle temporal gyrus, bilateral middle frontal gyri, right superior medial gyrus, left superior parietal gyrus and angular gyrus. The ICC maps underlying the presented results are provided on Neurovault.org (https://identifiers.org/neurovault.collection:9026).

#### Spearman’s rho

We computed spearman’s rho coefficients between the food category contrast maps and the neutral contrast maps to assess whether there is a high correlation between the constituting conditions (food and neutral), which would reduce the maximum possible reliability of the difference contrast (food-neutral), due to elimination of shared variance during performing the subtraction. Results demonstrate a substantial correlation between the all three food stimuli category contrast maps and the neutral stimuli contrast maps (rho_sweet-neutral_ = 0.49, SD = 0.29, *R*^2^ = 0.24, rho_low-neutral_ = 0.42, SD = 0.33, *R*^2^ = 0.17, rho_high-neutral_ = 0.42, SD = 0.33, *R*^2^ = 0.17). This indicates that both food and neutral conditions share about 17–24% of their variance. A part of this variance is removed by subtracting both conditions, which results in lower reliability of the difference contrast [23].

#### Similarity

**Fig. 2:**
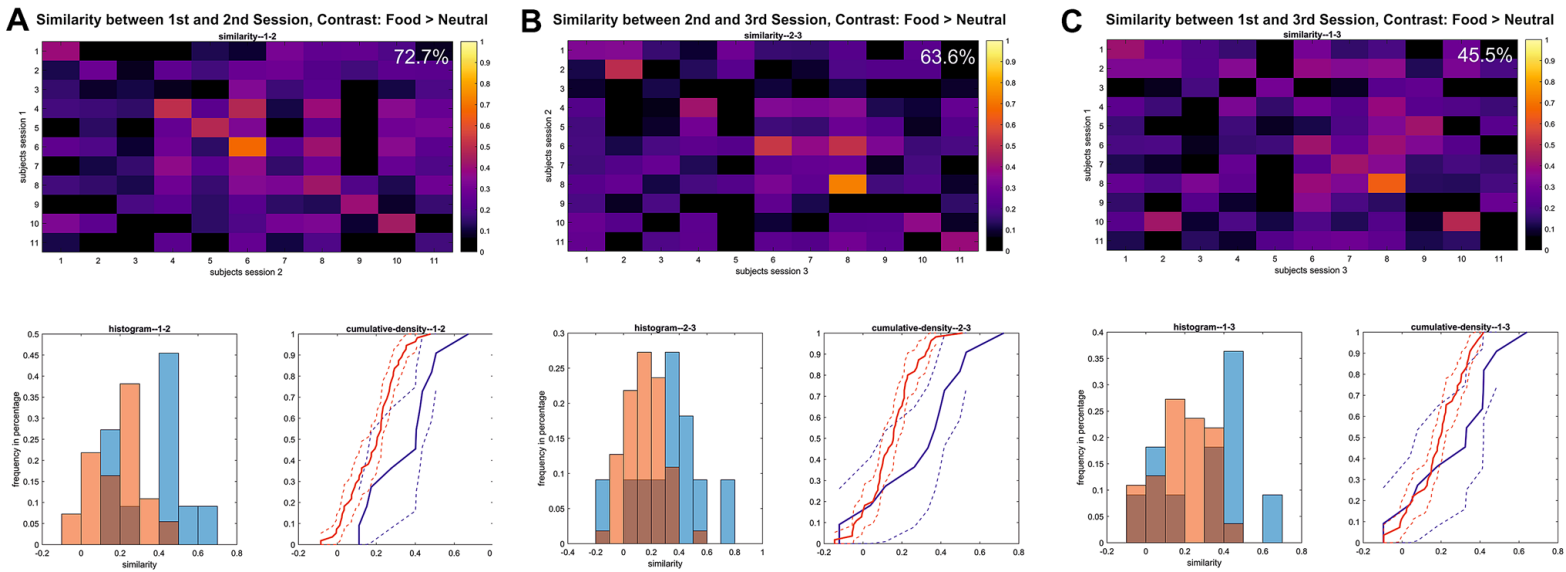
Similarity maps (upper row) and empirical cumulative distribution functions (lower row—red lines: between-subject similarity, blue lines: within-subject similarity) for the contrast food–neutral and comparisons between **a** 1st and 2nd fMRI session, **b** 2nd and 3rd fMRI session and **c** 1st and 3rd fMRI session. The diagonal of each color matrix represents the within-subject similarity values. Re-identification of a subject based on the neural activation map is affirmed the within-subject similarity value (diagonal) exceeds all between-subject association coefficients of the same participant (i.e. similarity values in the respective row of the matrix). Higher within-subject similarity is also illustrated by a right-shift of the cumulative density functions for the within-subject similarity values (blue lines) relative to the between-subject similarity (red lines). Percent values in the upper right of the upper row panels represent the number of individuals that could be identified based on their brain response (i.e. within-subject similarity values exceeded all between-subject similarity values for the respective participant [rows in matrix])

The analyses of similarity between activation maps for the difference contrast (food > neutral) showed a gradual decrease of within-subject similarity over comparisons between fMRI sessions with increasing time between the respective sessions (i.e. higher within-subject similarity between T0 and T2 that were 10 weeks apart vs. T2 and T3 that were 16 weeks apart). This reflected in lower *t* values for the comparisons between within-subject and between-subject similarity for the respective sessions and lower mean similarity values (*r*_T0_T1_ = 0.37, *t*_T0_T1_ = 5.14, *p* < 0.001, *r*_T1_T2_ = 0.32, *t*_T1_T2_ = 3.82, *p* < 0.05, *r*_T1_T3_ = 0.29 *t*_T1_T3_ = 3.01, *p* < 0.05). The difference between within and between-subject similarity is visible in the matrices and cumulative distribution functions for within- and between-subject similarity in Fig. 2. The proportion of patients that could be re-identified based on their neural brain activation (i.e. the magnitude of within-subject similarity exceeded all between-subject similarity values). While about 73% of the patients could be re-identified between T0 and T1, this number dropped when comparing longer time periods between T1–T2 (64%) and T0–T2 (45%, see Fig. 2).

## Discussion

The purpose of this study was to investigate the longitudinal reliability of the different task contrasts an established food cue-reactivity task. ICC values indicated good to excellent reliability of brain activation, captures by the common difference contrast food vs. neutral, in a range of brain areas (i.e. the mesolimbic system with putamen and caudate, as well as parts of the frontal and occipital cortices) over a time period of 26 weeks. In addition the reliability of food cue-induced brain activation in these brain regions, indexed by the difference contrast food vs. neutral, outperformed the reliability of subjective food craving (i.e. craving during food blocks vs. neutral blocks) that was measured concurrently during fMRI using visual analogue scales. Still, it should be noted that local reliability did not surpass the threshold for good reliability in all areas of the mesocorticolimbic system, which were implicated in processing food cues [33]. Furthermore, Jaccard and Dice coefficients, which provide estimates for the replicability of significant activation clusters across the whole brain, indicated that only a small proportion of activation could be replicated, when investigating the difference contrast (food > neutral). This stood in sharp contrast to the results for the constituting task contrast conditions food vs. baseline and neutral vs. baseline separately. For these two contrast conditions, Jaccard and Dice coefficients showed that more than 50% of the super-threshold clusters could be replicated during the other assessment sessions. This indicates that the global reliability of the common difference contrast food vs. neutral is limited. Several reasons might account for these findings. In previous studies, Infantolino and colleagues (2018) argued that the correlation between the constituting contrast conditions of a difference contrast place a limit on the reliability of the resulting difference measure, because in this case, large proportions of the shared and potentially true variance are eliminated by subtracting both constituting task conditions. The authors sustained their argument with data on the difference contrast between face- and shape-matching trials of a so-called faces paradigm, where the constitution shape and face conditions correlated to 0.97 [19, 23]. Other fMRI studies that also computed difference contrasts as the measure of interest, reported higher reliability of brain activation that was mirrored by an only modest correlation between the constituting conditions [30]. Current data show a moderate correlation between the food and neutral contrast images with a shared variance of about 24%. This supports the notion that the global reliability of the difference contrast (food vs. neutral) in the current dataset is limited by the correlation between the constituting conditions, which results in an elimination of proportions of the shared variance. The similarity analyses indicated that the capacity to identify individual individuals based on their individual brain activation pattern during the food vs. neutral contrast fades, when time periods between sessions increase. This was an expected finding and suggests that in the case of food cue-reactivity, follow-up fMRI scans should not be scheduled too far apart, when one intends to yield high reliability.

The only other previous study specifically investigating reliability of food cue-reactivity used a pre-selected range of ROIs (insula, putamen, amygdala, orbitofrontal cortex, caudate) and reported overall poor reliability in these ROIs. Several reasons might have accounted for the differences between this and the current study. The study by Drew Sayer et al. [9] used a different fMRI task design. The number of blocks of neutral and food stimuli per run was markedly lower (i.e. 3 and 3) compared to the task that was used as a basis for current analyses. Fewer data points per subject might, however, lead to less robust estimates of the individuals “true” mean value, e.g. brain response. Additionally, the study did not investigate voxel-wise reliability, but instead extracted brain activation estimates from predefined regions of interest and focused on the ICC as only an estimate for reliability. The use of the local maxima that were detected in the group level analyses as center of these ROIs, might have biased results. Studies have shown that a robust effect on the group level does not indicate stability or reliability of within-subject effects and might also be influenced by outliers [23]. Hence, the focus on these specific ROIs that only covered a diameter of 3 mm around the activation maximum, might have limited the possibility to identify regions with robust reliability. Current atlas-based summaries support the notion that the areas under investigation, specifically the caudate, putamen and amygdala show at least moderate reliability, when using the fMRI task of the current study.

Multiple studies intended to determine predictors for successful weight loss after bariatric surgery and establish neural “biomarkers” [21, 32]. As reliability is a prerequisite for any measure that could potentially serve as “biomarker”, current results could inform future studies and support the notion that neural responses to food cues in a selected range of brain areas might indeed meet the requirements for a potential predictor of treatment outcome.

## Strengths and limitations

We investigated a specific block-design food cue-reactivity task that was used and validated in previous work by our group [17]. Hence, our results may be generalized for food cue-reactivity tasks that incorporate different picture sets or a different task design. Still, the convergence of the different reliability estimates supports the robustness of the findings and the applied methods. We also acknowledge that other methods for the estimation of fMRI reliability exist (e.g. support vector machine learning) and might be informative. We investigated the reliability in a clinical population undergoing surgery. Due to the fact that reliability depends on the population under investigation, we argue that this approach complements the investigation of healthy reference samples to assess the potential of fMRI-based markers for application in clinical populations. Still, the investigation of healthy samples and individuals with obesity are essential to yield robust estimates of reliability of food cue-reactivity without potential bias and reduction in reliability due to surgical intervention or weight loss and improve the overall precision of reliability estimates. We, however, intended to provide a conservative estimate of the reliability of food cue-reactivity, because we acknowledge that statistical control might not be feasible and is also arbitrary to a certain extent (e.g. only controlling variables that show a significant effect of time in a respective trial would lead to differences between trials). This might lead to bias in the estimating of the reliability of food cue-reactivity. It could be argued that the inclusion of patients without any treatment might be favorable with regards to yielding optimal reliability. However, we strongly advocate for testing reliability under the conditions in which the actual task is applied. When intending to use neural brain response as biomarker for monitoring e.g. treatment response, reliability of this putative biomarker should be tested under the very same conditions. It should be noted that reliability estimates, which are based on small datasets, are prone to imprecision, due to large confidence intervals and high impact of single participant data, which also accounts for the presented dataset. The complementary whole-brain analyses that compared brain responses towards food cues between the different assessment session did not yield significance, when applying a stringent whole-brain correction for multiple testing. This result is unexpected and contrasts previous studies that showed longitudinal changes in brain response from before to after surgery [29, 46]. The lack of significant main effects of time on brain response might relate to a limited power and a stringent whole-brain threshold (e.g. previous studies applied regions of interest analyses), resulting from the small dataset. However, power analyses indicated that analyses comparing different time points yielded sufficient power (see Supplementary Figure S1). Additionally, several significant findings were derived from studies applying more liberal regions of interest analyses. Overall, the lack of substantial time effects on the extent of food cue-induced brain response in the current dataset support the notion that reliability estimates were not substantially biased by surgical intervention.

## Conclusion

We could show excellent local longitudinal reliability in a range of brain areas of the reward (e.g. caudate, putamen) and food-cue-processing networks (e.g. occipital and frontal cortices) in participants with obesity from 2 weeks before, to 24 weeks after surgery. The reliability of food cue-reactivity in these areas outperformed to reliability of subjective craving measures that were measured concurrently. Our results suggest that fMRI-based measures might indeed be suitable to monitor and predict treatment outcome in participants with obesity undergoing bariatric surgery.

## Supplementary Information

Below is the link to the electronic supplementary material.

Supplementary file1 (DOCX 224 KB)
