# Supplementary material for: Reliability of neural food cue-reactivity in participants with obesity undergoing bariatric surgery: a 26-week longitudinal fMRI study
Source: Eur Arch Psychiatry Clin Neurosci. 2020 Dec 17;271(5):951–62. doi: 10.1007/s00406-020-01218-8 (PMC8236041; doi:10.1007/s00406-020-01218-8)

**Supplementary Table S1.** Atlas-based mean intraclass correlation (ICC) values for the N=120 anatomical regions specified in the automated anatomical labeling (aal) atlas (contrast: food > neutral stimuli, comparisons across sessions 1 to 3). Regions exceeding a mean ICC value of 0.4, corresponding to a moderate reliability, are marked in bold font.

|  | Region (AAL) | Mean ICC value |
| --- | --- | --- |
|  | **Amygdala_L** | **0.40763759** |
|  | Amygdala_R | 0.36786118 |
|  | **Angular_L** | **0.4153282** |
|  | **Angular_R** | **0.48685384** |
|  | **Calcarine_L** | **0.53597925** |
|  | **Calcarine_R** | **0.57968044** |
|  | **Caudate_L** | **0.48102494** |
|  | **Caudate_R** | **0.44163647** |
|  | Cerebelum_10_L | 0.27364602 |
|  | Cerebelum_10_R | 0.09230089 |
|  | Cerebelum_3_L | 0.06165687 |
|  | Cerebelum_3_R | 0.17436859 |
|  | Cerebelum_4_5_L | 0.15992892 |
|  | Cerebelum_4_5_R | 0.21595067 |
|  | Cerebelum_6_L | 0.30154894 |
|  | Cerebelum_6_R | 0.32449259 |
|  | Cerebelum_7b_L | 0.00027952 |
|  | Cerebelum_7b_R | 0.15759062 |
|  | Cerebelum_8_L | -0.02479433 |
|  | Cerebelum_8_R | 0.10007577 |
|  | Cerebelum_9_L | 0.12451792 |
|  | Cerebelum_9_R | 0.06073853 |
|  | Cerebelum_Crus1_L | 0.34667738 |
|  | Cerebelum_Crus1_R | 0.34672601 |
|  | Cerebelum_Crus2_L | 0.16982958 |
|  | Cerebelum_Crus2_R | 0.26460454 |
|  | Cingulate_Ant_L | 0.31521474 |
|  | Cingulate_Ant_R | 0.29600421 |
|  | Cingulate_Mid_L | 0.27712185 |
|  | Cingulate_Mid_R | 0.27994037 |
|  | **Cingulate_Post_L** | **0.50434102** |
|  | **Cingulate_Post_R** | **0.49553011** |
|  | **Cuneus_L** | **0.56512844** |
|  | **Cuneus_R** | **0.5228328** |
|  | Frontal_Inf_Oper_L | 0.14184759 |
|  | Frontal_Inf_Oper_R | 0.21942506 |
|  | Frontal_Inf_Orb_2_L | 0.27682627 |
|  | Frontal_Inf_Orb_2_R | 0.21755721 |
|  | Frontal_Inf_Tri_L | 0.30462771 |
|  | Frontal_Inf_Tri_R | 0.24102302 |
|  | Frontal_Med_Orb_L | 0.25330428 |
|  | Frontal_Med_Orb_R | 0.25289913 |
|  | **Frontal_Mid_2_L** | **0.41863018** |
|  | **Frontal_Mid_2_R** | **0.4209279** |
|  | Frontal_Sup_2_L | 0.32470793 |
|  | Frontal_Sup_2_R | 0.32815861 |
|  | **Frontal_Sup_Medial_L** | **0.45450867** |
|  | **Frontal_Sup_Medial_R** | **0.40819146** |
|  | **Fusiform_L** | **0.54252807** |
|  | **Fusiform_R** | **0.58699878** |
|  | Heschl_L | 0.05446871 |
|  | Heschl_R | 0.20525357 |
|  | Hippocampus_L | 0.30091927 |
|  | Hippocampus_R | 0.18147327 |
|  | Insula_L | 0.20153209 |
|  | Insula_R | 0.1647087 |
|  | **Lingual_L** | **0.6225418** |
|  | **Lingual_R** | **0.57012037** |
|  | **Occipital_Inf_L** | **0.68905961** |
|  | **Occipital_Inf_R** | **0.63069714** |
|  | **Occipital_Mid_L** | **0.70694477** |
|  | **Occipital_Mid_R** | **0.73225597** |
|  | **Occipital_Sup_L** | **0.6863284** |
|  | **Occipital_Sup_R** | **0.64287727** |
|  | OFCant_L | 0.20520715 |
|  | OFCant_R | 0.26953573 |
|  | OFClat_L | 0.11979768 |
|  | OFClat_R | 0.31150854 |
|  | OFCmed_L | 0.10423343 |
|  | OFCmed_R | 0.18515158 |
|  | OFCpost_L | 0.30889935 |
|  | OFCpost_R | 0.24117885 |
|  | Olfactory_L | 0.24754212 |
|  | Olfactory_R | 0.26154753 |
|  | Pallidum_L | 0.27670088 |
|  | Pallidum_R | 0.21066503 |
|  | Paracentral_Lobule_L | 0.14723272 |
|  | Paracentral_Lobule_R | 0.06484738 |
|  | ParaHippocampal_L | 0.30799088 |
|  | ParaHippocampal_R | 0.28349796 |
|  | Parietal_Inf_L | 0.26225495 |
|  | Parietal_Inf_R | 0.22906141 |
|  | **Parietal_Sup_L** | **0.41622173** |
|  | Parietal_Sup_R | 0.3988176 |
|  | Postcentral_L | 0.16078831 |
|  | Postcentral_R | 0.08968218 |
|  | Precentral_L | 0.39168091 |
|  | Precentral_R | 0.18921363 |
|  | Precuneus_L | 0.36465171 |
|  | Precuneus_R | 0.35655186 |
|  | **Putamen_L** | **0.45442722** |
|  | Putamen_R | 0.30970413 |
|  | Rectus_L | 0.17306424 |
|  | Rectus_R | 0.14920027 |
|  | Rolandic_Oper_L | 0.13431982 |
|  | Rolandic_Oper_R | 0.10625991 |
|  | Supp_Motor_Area_L | 0.26899723 |
|  | Supp_Motor_Area_R | 0.18813331 |
|  | SupraMarginal_L | 0.05553424 |
|  | SupraMarginal_R | 0.36478604 |
|  | Temporal_Inf_L | 0.35265871 |
|  | Temporal_Inf_R | 0.34174827 |
|  | Temporal_Mid_L | 0.2828941 |
|  | **Temporal_Mid_R** | **0.46616954** |
|  | Temporal_Pole_Mid_L | 0.17525254 |
|  | Temporal_Pole_Mid_R | 0.23368946 |
|  | Temporal_Pole_Sup_L | 0.2615089 |
|  | Temporal_Pole_Sup_R | 0.09053107 |
|  | Temporal_Sup_L | 0.18427253 |
|  | Temporal_Sup_R | 0.34549048 |
|  | Thalamus_L | 0.23469258 |
|  | Thalamus_R | 0.23898485 |
|  | Vermis_1_2 | 0.19670743 |
|  | Vermis_10 | 0.25951009 |
|  | Vermis_3 | 0.14516806 |
|  | Vermis_4_5 | -0.03299619 |
|  | Vermis_6 | 0.12162924 |
|  | Vermis_7 | -0.00733943 |
|  | Vermis_8 | -0.18407945 |
|  | Vermis_9 | -0.06657724 |

**Supplementary Figure S1.** Depiction of the course of mean craving ratings for food stimuli for the three assessment sessions, for a) the 1^st^, b) the 2^nd^ and c) the 3^rd^ assessment. Depiction of the course of mean craving ratings for neutral stimuli for the three assessment sessions, for d) the 1^st^, e) the 2^nd^ and f) the 3^rd^ assessment (Mean + 2 SE).

**
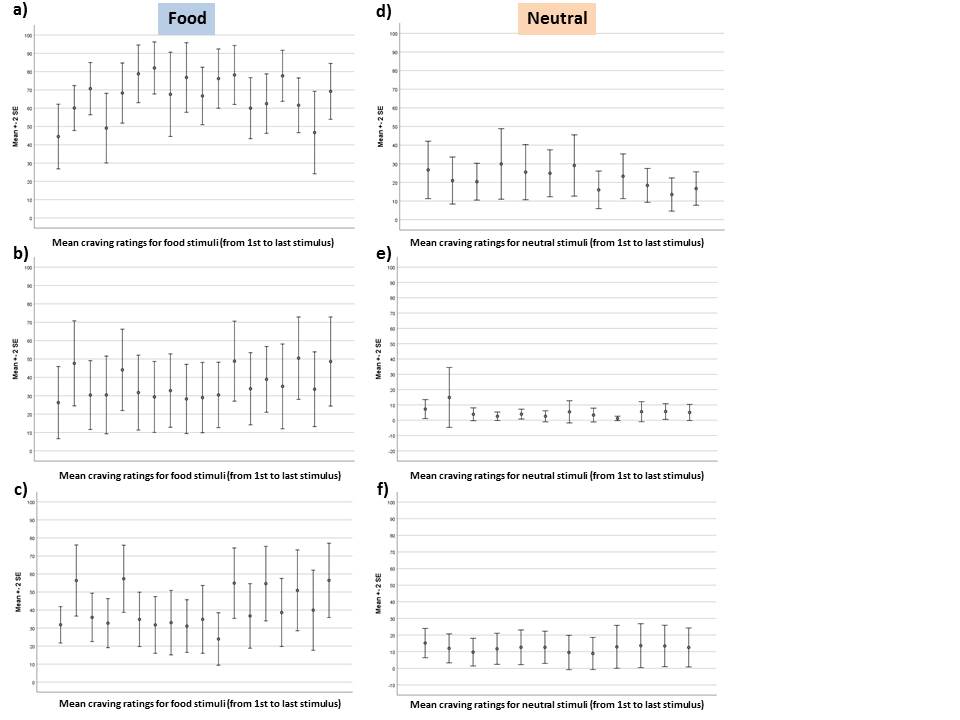
**

**Supplementary Figure S2.** Depiction of power estimates and mean effects for different brain regions defined by the automated anatomical labeling (aal) atlas, which were computed for a pairwise comparison between 1^st^ and 3^rd^ assessment sessions, based on the dataset of N=11 participants, using the FMRIpower software toolbox for SPM (<https://www.nitrc.org/projects/fmripower/>).


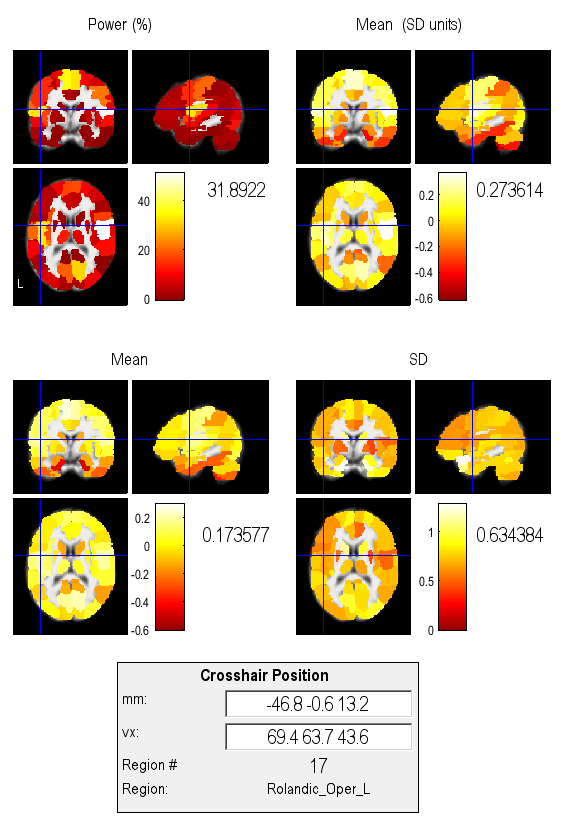

Supplement: Supplementary file 1 — Supplementary file1 (DOCX 224 KB) [file 406_2020_1218_MOESM1_ESM.docx]
